# Enhancing Chemotherapy Response with Bmi-1 Silencing in Ovarian Cancer

**DOI:** 10.1371/journal.pone.0017918

**Published:** 2011-03-21

**Authors:** Enfeng Wang, Sanjib Bhattacharyya, Annamaria Szabolcs, Cristian Rodriguez-Aguayo, Nicholas B. Jennings, Gabriel Lopez-Berestein, Priyabrata Mukherjee, Anil K. Sood, Resham Bhattacharya

**Affiliations:** 1 Department of Biochemistry and Molecular Biology, Mayo Clinic College of Medicine, Rochester, Minnesota, United States of America; 2 Department of Gynecologic Oncology, M. D. Anderson Cancer Center, Houston, Texas, United States of America; 3 Department of Cancer Biology, M. D. Anderson Cancer Center, Houston, Texas, United States of America; 4 Center for RNA Interference and Non-Coding RNA, M. D. Anderson Cancer Center, Houston, Texas, United States of America; 5 Department of Experimental Therapeutics, M. D. Anderson Cancer Center, Houston, Texas, United States of America; Ohio State University, United States of America

## Abstract

Undoubtedly ovarian cancer is a vexing, incurable disease for patients with recurrent cancer and therapeutic options are limited. Although the polycomb group gene, *Bmi-1* that regulates the self-renewal of normal stem and progenitor cells has been implicated in the pathogenesis of many human malignancies, yet a role for Bmi-1 in influencing chemotherapy response has not been addressed before. Here we demonstrate that silencing Bmi-1 reduces intracellular GSH levels and thereby sensitizes chemoresistant ovarian cancer cells to chemotherapeutics such as cisplatin. By exacerbating ROS production in response to cisplatin, Bmi-1 silencing activates the DNA damage response pathway, caspases and cleaves PARP resulting in the induction apoptosis in ovarian cancer cells. In an *in vivo* orthotopic mouse model of chemoresistant ovarian cancer, knockdown of Bmi-1 by nanoliposomal delivery significantly inhibits tumor growth. While cisplatin monotherapy was inactive, combination of Bmi-1 silencing along with cisplatin almost completely abrogated ovarian tumor growth. Collectively these findings establish Bmi-1 as an important new target for therapy in chemoresistant ovarian cancer.

## Introduction

Ovarian cancer has the highest mortality rate among all gynecologic malignancies [1]. Despite initial response to surgical debulking and front-line platinum/taxane chemotherapy, most tumors eventually develop a drug resistant relapse [2], [3]. Evidence suggests that cisplatin resistance might be the result of a defective apoptotic program. In this case, increased levels of DNA damage would be required to induce the signal initiating apoptosis [4], [5].

*Bmi-1*, a polycomb group gene, regulates the proliferative activity of normal stem and progenitor cells [6]. It is also indispensable for the self-renewal of neural [7], [8] and haematopoietic stem cells [9]. Bmi-1 is frequently upregulated in a variety of cancers including ovarian cancer and its correlation with clinical grade/stage, lymph node metastasis and poor prognosis has been reported [10], [11], [12], [13], [14], [15], [16], [17], [18], [19]. *Bmi-1* causes neoplastic transformation of lymphocytes and co-operates with H-Ras giving rise to metastatic breast cancer in mice [20], [21], [22], all strongly suggesting an oncogenic role in epithelial malignancies. In addition, isolated ovarian cancer stem cells exhibit much higher Bmi-1 levels compared to the differentiated or parental bulk tumor cells and have increased resistance to cisplatin and paclitaxel when compared to the tumor cells [23]. Also the increased expression of Bmi-1 was one of the key regulatory factors determining a cellular phenotype captured by the expression of a death-from-cancer signature in a broad spectrum of therapy-resistant clinically lethal malignancies [24]. Despite this wealth of information a possible role for Bmi-1 in influencing chemotherapy response has not been addressed before. In this context, determining the mechanism by which Bmi-1 silencing sensitizes the cancer cells to cisplatin would be important for development of new therapeutic strategies to combat ovarian cancer.

The most active chemotherapy agents in ovarian cancer are the platinum analogues, cisplatin and carboplatin. The antitumor activity of cisplatin (cis-diamminedichloroplatinum (II) was discovered by Rosenberg and colleagues in 1961 [25]. Cisplatin has been the most active drug for the treatment of ovarian cancer for the last 4 decades and the prognosis for women with ovarian cancer can be defined by the tumor response to cisplatin [26]. Although the majority of patients with ovarian cancer respond to front-line platinum combination chemotherapy the majority will develop disease that becomes resistant to cisplatin and will ultimately succumb to the disease [26]. Thus methods of preventing or overcoming resistance to cisplatin could have a major impact in the fight against this disease.

Here we demonstrate that *Bmi-1* plays an important role in sensitization of chemoresistant ovarian cancer cells to cisplatin. We also show that this sensitization is through a novel pathway modulated by Bmi-1, namely reactive oxygen species (ROS) induction causing engagement of the DNA damage response (DDR) pathway leading to apoptosis. We also establish Bmi-1 as a valid therapeutic target *in vivo* using a readily translatable approach of nanoliposomal delivery of siRNA into an orthotopic mouse model of ovarian cancer.

## Results

### Knockdown of Bmi-1 enhances cisplatin sensitivity in vitro

**Figure 1:**
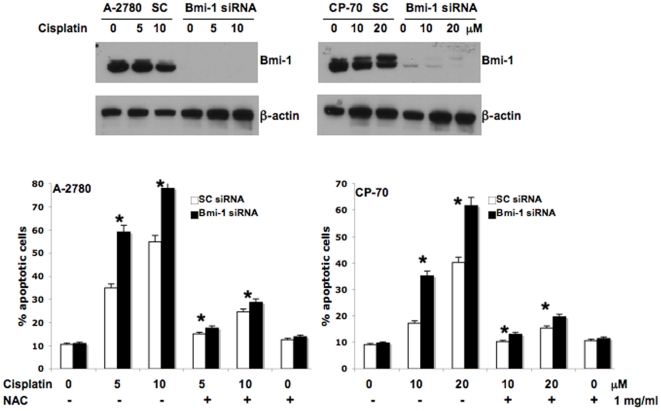
Bmi-1 knockdown sensitizes ovarian cancer cells to cisplatin through ROS pathway. The top panel demonstrates efficient knockdown of Bmi-1 by siRNA in the ovarian cancer cell lines (SC = scrambled control siRNA) as determined by Western blot. The bottom panel demonstrates percent apoptosis as determined by Annexin/FITC-PI staining. Ovarian cancer cells transfected with scrambled control or Bmi-1 siRNA were treated with or without cisplatin for 48 h. NAC pre-treatment was done for 1 h before addition of cisplatin where appropriate.

We have previously shown that reduction of Bmi-1 protein levels in ovarian cancer cells using microRNA 15a/16 decreases clonal growth and proliferation [27]. Here, we wanted to test if knockdown of Bmi-1 affected cisplatin mediated apoptosis in ovarian cancer cells. Efficient knockdown of Bmi-1 in A-2780 and CP-70 cells was confirmed by comparing with the scrambled control transfected cells after 48 h (Fig. 1). The siRNA transfected cells were treated with cisplatin for 48 h and apoptosis determined by the Annexin/FITC method. Apoptosis in the chemosensitive A-2780 control siRNA transfected cells was ∼35 and 55% when treated with 5 or 10 uM cisplatin alone respectively. In contrast, apoptosis in the chemoresistant CP-70 was ∼17 and 40% when treated with 10 or 20 uM cisplatin alone respectively indicating resistance of these cells towards cisplatin induced apoptosis (Fig. 1). Importantly treating the Bmi-1 silenced cells with cisplatin consistently enhanced apoptosis in both the cell lines by ∼15–20% (Fig. 1). For both the cell lines, the basal level of apoptosis determined without any treatment was ∼10%. Apoptosis experiments with three additional cell lines such as OVCAR-5, OV-202 and OV-167 yielded similar results (data not shown). These data confirmed that knockdown of Bmi-1 could sensitize ovarian cancer cells to cisplatin induced cell death.

Recently, it has been reported that neurons, thymocytes and bone marrow cells isolated from Bmi-1 null mice have increased ROS levels than their wild-type counterparts [28], [29]. Furthermore, studies have reported the involvement of ROS generation in cisplatin-mediated apoptosis [30], [31]. Hence, to determine how silencing of Bmi-1 sensitizes drug-resistant ovarian cancer cells to cisplatin induced apoptosis, we investigated the possible involvement of ROS. Therefore, Bmi-1 or scrambled-control siRNA transfected ovarian cancer cells were pre-treated with N-Acetyl Cysteine (NAC) at 1 mg/ml for 1 h followed by cisplatin treatment for 48 h. Significant inhibition of cisplatin mediated apoptosis in both control and Bmi-1 knockdown cells was observed in the presence of ROS scavenger NAC (Fig. 1). These data indicate that the augmented apoptosis observed in the cisplatin treated Bmi-1 silenced cells was due to the involvement of ROS and led us to determine ROS production as a next logical step.

### Knockdown of Bmi-1 increases cisplatin-mediated ROS production

**Figure 2:**
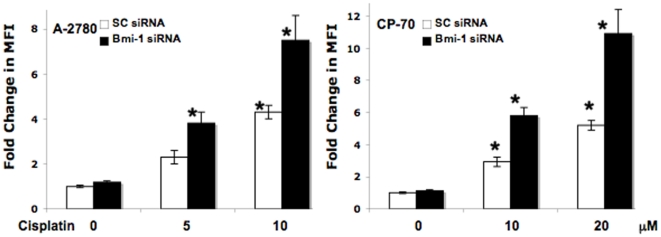
Bmi-1 knockdown increases cisplatin-mediated ROS production in ovarian cancer cells. Ovarian cancer cells transfected with scrambled control or Bmi-1 siRNA were treated with or without cisplatin for 24 h. Subsequently the cells were incubated with 5 µM carboxy-H2DCFDA in fresh HBSS for 30 min at 37°C. The cells were harvested with trypsin and fluorescence of the labeled cells was measured at an excitation wavelength of 485 nm and emission wavelength of 530 nm by using Fluorolog 3 (Jobin-Yvon Horiba). Ratio of mean fluorescence intensity (MFI) with respect to the untreated scrambled control is represented.

We next determined ROS production in scrambled control or Bmi-1 siRNA transfected cells treated with or without cisplatin by measuring fluorescence using DCFDA. Cisplatin treatment alone significantly increased ROS generation at 10 uM in A-2780 cells and at 10 and 20 uM in CP-70 cells. In both the cell lines, knockdown of Bmi-1 followed by any cisplatin treatment significantly increased ROS generation (Fig. 2). These data corroborate our previous observations on apoptosis and posits ROS as the primary reason for enhanced sensitization of ovarian cancer cells to cisplatin. In order to determine a cause for the exacerbated ROS mediated apoptosis observed we next determined total cellular glutathione (GSH) levels.

### Bmi-1 regulates GSH production

**Figure 3:**
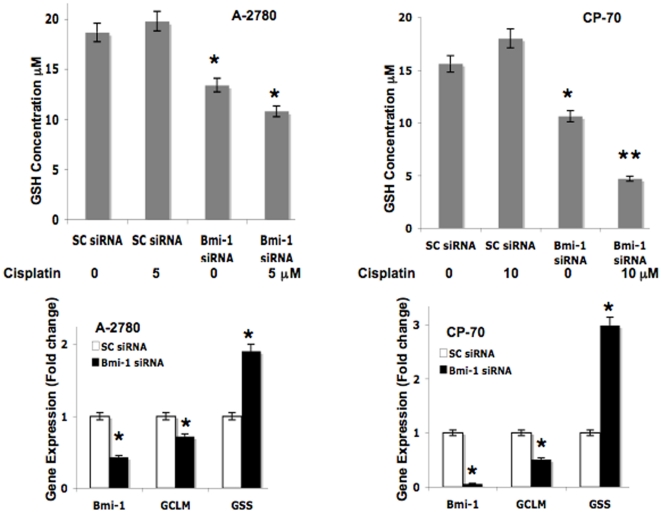
Bmi-1 knockdown perturbs the GSH biosynthesis pathway. The top panel represents total cellular GSH measured (Materials and methods) in ovarian cancer cells transfected with scrambled control or Bmi-1 siRNA treated with or without cisplatin for 24 h. The bottom panel represents fold change in gene expression (normalized with beta actin and compared to scrambled control) as determined by quantitative RT-PCR of ovarian cancer cells transfected with scrambled control or Bmi-1 siRNA for 48 h.

Reduced GSH is a critical component of the cell's antioxidant network, being directly involved in scavenging ROS and in maintaining thiol proteins in their reduced state [32], [33], [34]. Thus to determine a cause for the increased ROS mediated apoptosis observed in Bmi-1 silenced cells, we next determined total intracellular GSH levels. Lysates were prepared from scrambled control or Bmi-1 siRNA transfected cells treated with or without cisplatin. Knockdown of Bmi-1 alone significantly decreased cellular GSH levels (∼30% in A-2780 and ∼40% in CP-70) and this was further decreased when combined with cisplatin treatment (∼45% in A-2780 and ∼56% in CP-70) (Fig. 3). Cisplatin treatment alone however had no effect on GSH levels. These data suggests that at least some of the oxidative stress mediated effects observed in the Bmi-1 knockdown ovarian cancer cells is due to decreased intracellular GSH levels.

To further investigate the cause of reduced GSH content in Bmi-1 silenced cells we next determined whether the expression levels of the key enzymes involved in the GSH biosynthesis pathway were altered. We performed quantitative RT-PCR to determine the gene expression level of glutamate-cysteine ligase (GCLM) and glutathione synthase (GSS), in the Bmi-1 siRNA transfected ovarian cancer cell lines. Glutamate-cysteine ligase (GCLM) is the first rate-limiting enzyme of the glutathione biosynthesis pathway [35]. In the second step, glutathione synthase (GSS), converts gamma-L-glutamyl-L-cysteine to glutathione [35]. While mRNA expression of Bmi-1 was reduced as expected in Bmi-1 silenced cells, interestingly mRNA expression of GCLM significantly decreased while that of GSS increased (Fig. 3). The trend was similar in both ovarian cancer cell lines. These results suggest that perturbation of GCLM, the rate-limiting enzyme is sufficient to reduce total cellular GSH levels and subsequent fold increase in GSS mRNA levels probably represent a defense mechanism for the cells trying to relieve oxidative stress.

### Bmi-1 modulates the DDR pathway

**Figure 4:**
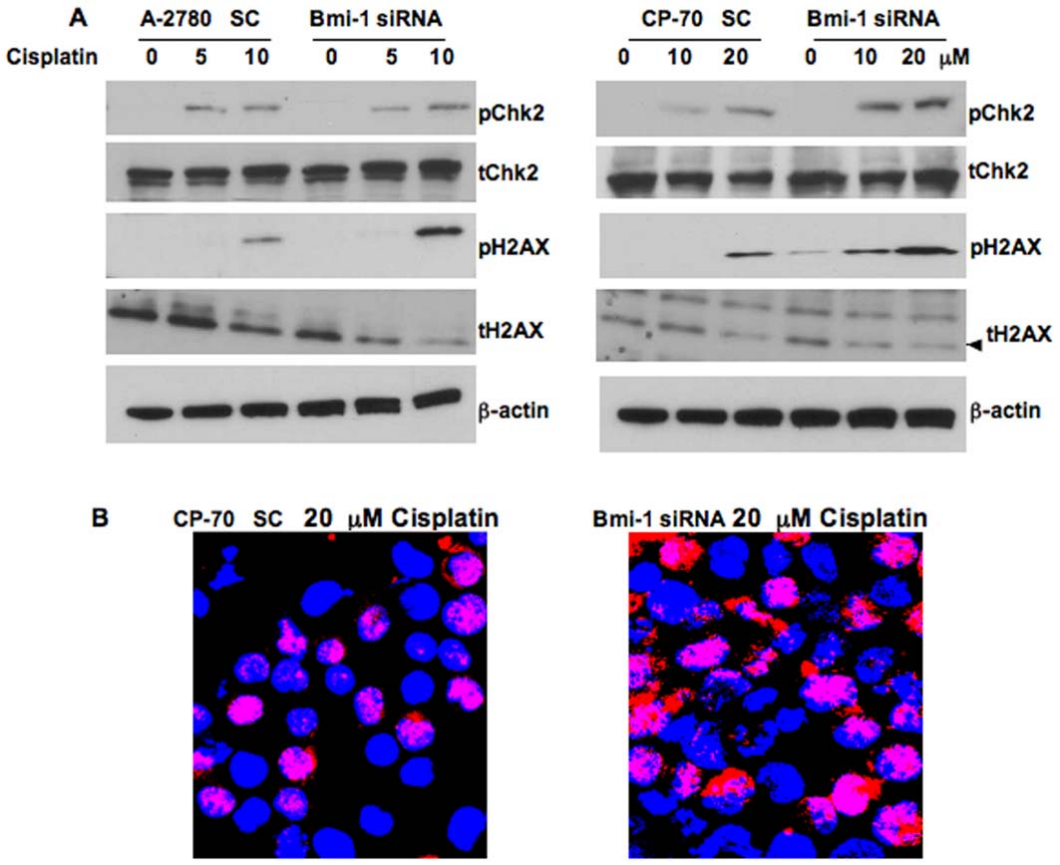
Bmi-1 knockdown augments engagement of the DDR pathway in cisplatin treated ovarian cancer cells. (A) Ovarian cancer cells transfected with scrambled control or Bmi-1 siRNA were treated with or without cisplatin for 48 h. Western blot was performed for phospho Chk-2, total Chk-2, phospho-H2AX, total H2AX and beta actin using respective antibodies. (B) Scrambled control or Bmi-1 siRNA transfected CP-70 cells were subjected to confocal microscopy using 53BP1 antibody (red) and DAPI (blue nuclear staining) to demonstrate nuclear foci formation.

To further investigate the mechanism of ROS mediated enhanced apoptosis by cisplatin in Bmi-1 silenced ovarian cancer cells we wanted to test the involvement of the DNA damage and repair (DDR) pathway. Previous studies have reported that oxidative stress can trigger activation of the DDR pathway [36]. In order to test this we determined phosphorylation levels of Chk2 and H2AX two important biomarkers for DNA damage and activation of the DDR pathway [37]. Cisplatin treatment alone induced phosphorylation of Chk2 and H2AX in a concentration dependent manner and knockdown of Bmi-1 further exacerbated this effect (Fig. 4A). In corroboration, increased nuclear foci formation was observed by 53 BP1 immunoflourescence in CP-70 Bmi-1 knockdown cells treated with cisplatin (Fig. 4B). These results indicate that sustained levels of ROS generated upon Bmi-1 knockdown coupled with cisplatin treatment are sufficient to directly damage the DNA and engage the DDR pathway. A large body of literature supports that DNA damage can lead to apoptosis via activation of caspases [38], [39]. Therefore we next proceeded to determine activation of caspases in Bmi-1 silenced ovarian cancer cells treated with or without cisplatin.

### Bmi-1 regulates effectors of apoptosis

**Figure 5:**
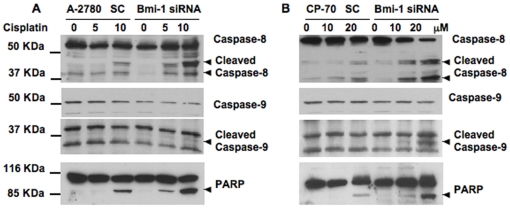
Effect of Bmi-1 knockdown on apoptotic markers. Ovarian cancer cells transfected with scrambled control or Bmi-1 siRNA were treated with or without cisplatin for 48 h. Western blot was performed for caspase-8, caspase-9 and PARP using respective antibodies.

Established mediators of apoptosis include caspases [40]. We next determined cleavage of caspase 8 and caspase 9 in control scrambled or Bmi-1 siRNA transfected cells treated with or without cisplatin. Cisplatin treatment alone caused cleavage of caspase 8 but not caspase 9. It is plausible that with higher concentrations of cisplatin cleavage of caspase 9 could be observed. Importantly however, combination of Bmi-1 knockdown and cisplatin treatment significantly and dose dependently increased cleavage of caspase 8 and caspase 9 in both the cell lines (Fig. 5).

PARP is one of the main cleavage targets of activated caspases and cleaved PARP facilitates cellular disassembly and serves as a marker of cells undergoing apoptosis [40]. In Bmi-1 knockdown cells, clear cleavage of PARP was observed in a cisplatin dose dependent manner (Fig. 5). All of these *in vitro* data indicate that cisplatin treatment of Bmi-1 knockdown cells enhances apoptosis by increasing ROS production, causing engagement of the DDR pathway and activating caspases. We next proceeded to determine the therapeutic efficacy of silencing Bmi-1 in an orthotopic chemoresistant mouse model of ovarian cancer.

### Knockdown of Bmi-1 enhances cisplatin sensitivity in vivo

**Figure 6:**
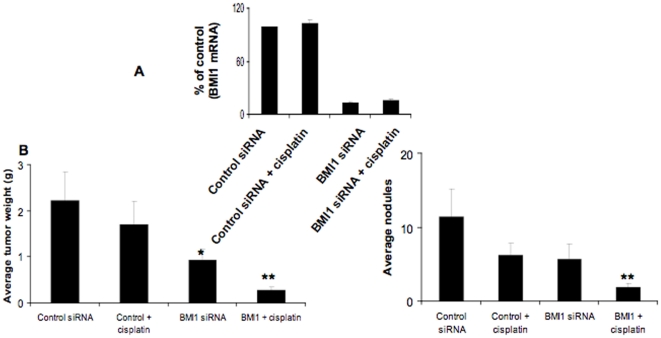
Effect of Bmi-1 knockdown on orthotopic chemoresistant ovarian cancer growth. To assess the effects of siRNA therapy on tumor growth, treatment was initiated 1 wk after i.p. injection (1.0×10^6^ CP20) of tumor cells. Mice were divided into four groups (n = 10 mice per group): (a) control siRNA-DOPC (150 µg/kg i.p. twice weekly), (b) control siRNA-DOPC + cisplatin (160 µg/mouse i.p. weekly), (c) Bmi-1 siRNA-DOPC (150 µg/kg i.p. twice weekly), and (d) Bmi-1 siRNA-DOPC + cisplatin (doses same as individual treatments). Treatment was continued until 4 weeks after tumor inoculation before sacrifice. (A) Total RNA was isolated from a portion of the tumor tissues and subjected to RT-PCR using primers for Bmi-1 and beta actin. The comparative C_t_ method was used to calculate the relative abundance of mRNA compared with that of beta actin expression. The experiment was performed in triplicate and significance determined using two-sided Student's t test, P<0.05 was considered significant. (B) Mouse and tumor weights and (C) the number of tumor nodules for each group were compared using Student's t test (for comparisons of two groups). A two-tailed P≤0.05 was deemed statistically significant.

Next the therapeutic potential of Bmi-1 was tested by *in vivo* delivery of Bmi-1 siRNA using DOPC (1,2-dioleoyl-sn-glycero-3-phosphatidylcholine) nanoliposomes in the orthotopic CP-20 mouse model. This method of delivery has been extensively characterized previously for duration of knockdown and has been shown to lack non-specific inflammatory responses [41], [42]. To simulate treatment of advanced small-volume disease, therapy was initiated 1 week after tumor cell injection. Mice were divided in to the following four groups (n = 10 mice per group): (a) control siRNA-DOPC (150 µg/kg i.p. twice weekly), (b) control siRNA-DOPC + cisplatin (160 µg/mouse i.p. weekly), (c) Bmi-1 siRNA-DOPC (150 µg/kg i.p. twice weekly), and (d) Bmi-1 siRNA-DOPC + cisplatin (doses same as individual treatments). All of the animals were sacrificed after 4 weeks of therapy. Efficient knockdown of Bmi-1 (∼85%) was first confirmed by RT-PCR (Fig. 6A). Treatment with Bmi-1 siRNA alone resulted in significant (∼60%) reduction in tumor weight compared to the control siRNA group. Combination therapy with Bmi-1 siRNA and cisplatin resulted in even greater (∼80%) reduction in tumor weight compared to the cisplatin only treated group (Fig. 6B). To further evaluate this effect, the number of tumor nodules formed in each group was determined. Again combination therapy showed greatest effect with ∼70% fewer tumor nodules compared to the cisplatin only treated group (Fig. 6B). No obvious toxicity was noted in the animals during therapy experiments as assessed by changes in behavior, feeding habits, and mobility. The mean body weight was also similar between the treatment groups (data not shown).

### Effect of Bmi-1 knockdown on proliferation and apoptosis in vivo

**Figure 7:**
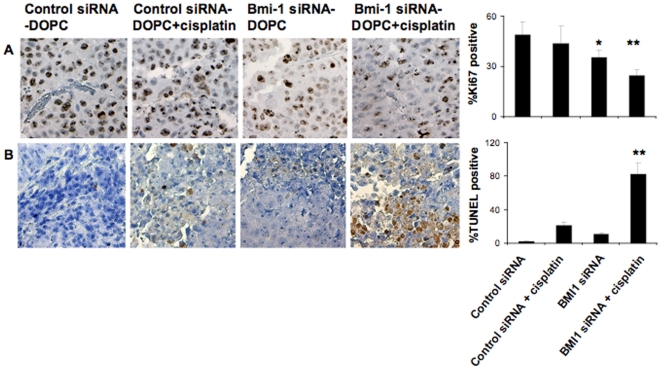
Knockdown of Bmi-1 leads to decreased proliferation and increased apoptosis of ovarian tumor *in vivo*. (A) Immunohistochemical staining for Ki67 and (B) TUNEL was conducted to assess cell proliferation and apoptosis. Original magnification 200X. Quantification is shown graphically on the right. Treatment arms were compared using Student's t test and P≤0.05 was deemed statistically significant.

To corroborate our *in vitro* data we next examined the effect of Bmi-1 knockdown on *in vivo* tumor cell proliferation and apoptosis by using Ki67 and TUNEL staining. Combination therapy with Bmi-1 siRNA and cisplatin showed the greatest effect with ∼50% decrease in proliferation compared to the control untreated group (Fig. 7). Similarly, an approximately 80% increase in apoptosis was observed in the combination therapy group compared to the control treated group (Fig. 7). Therefore we demonstrate that silencing of Bmi-1 in ovarian cancer cells whether *in vitro* or *in vivo* increases apoptosis in response to cisplatin.

## Discussion

Undoubtedly ovarian cancer is a vexing, incurable disease for patients with recurrent cancer and therapeutic options are limited [1], [43]. Here we demonstrate Bmi-1 gene silencing as an effective option, which in combination with cisplatin enhances therapeutic efficacy even further. Moreover we delineate the mechanism of increased sensitivity to be primarily through ROS production. Frontline chemotherapy for ovarian cancer involves use of the platinum/taxane regimen, which are known to induce ROS production. We posit that knockdown of Bmi-1 will prove efficacious in combination therapy with this regimen. In this context a recent report has demonstrated Bmi-1 to be recruited early to the double-strand break site upon ionizing radiation damage [44]. Our results corroborate and extend this idea and demonstrate *in vitro* and *in vivo* that Bmi-1 silencing synergizes with increased oxidative stress leading to accumulation of DNA damage and apoptosis.

At least two previous publications demonstrated increased ROS levels in neurons, thymocytes and bone marrow cells isolated from Bmi-1 null mice [28], [29]. However the cause for this ROS production has been varyingly ascribed to p53 dependent and independent repression of anti-oxidant gene expression or impaired mitochondrial energetics [28], [29]. We show here that in addition to these pathways Bmi-1 also controls cellular GSH levels by regulating transcription of the enzymes involved in the GSH biosynthesis pathway. Notably transcription of GCLM is positively regulated by transcription factors such as Nrf-1 and NFκB. In the context of Bmi-1 knockdown, both of these transcription factors are downregulated in neurons and glioma cells respectively [28], [45]. Therefore it is possible that Bmi-1 silencing leads to downregulation of Nrf-1 and or NFκB in ovarian cancer cells, thereby decreasing transcription of GCLM resulting in reduced GSH synthesis. Importantly here we show that Bmi-1 by regulating ROS and GSH levels protects the ovarian cancer cells from chemotherapeutic insults.

The DNA damage response pathway can be activated by genotoxic stress such as those caused by chemotherapeutics and oxidative DNA damage. Indeed activation of DDR can lead to a pause in cell cycle progression, senescence or apoptosis. In accordance we find that the dual insult of oxidative damage caused by Bmi-1 knockdown and cisplatin treatment, which is known to cause double strand breaks in the DNA along with ROS production tips the threshold for ovarian cancer cells towards apoptosis by inducing phosphorylation of i) Chk2 and H2AX, ii) causing nuclear foci formation by 53BP1 and cleavage of apoptotic markers such as iii) caspases and PARP.

As we have demonstrated in our *in vivo* experiments, knockdown of Bmi-1 could be useful in clinical settings due to the following reasons; a) downregulation of Bmi-1 enhances cisplatin-induced apoptosis in ovarian cancer cells. b) Bmi-1 is required for self-renewal and maintenance of stem cells including ovarian cancer stem cells which by definition are resistant to chemotherapeutics [23]. c) Bmi-1 regulates multiple pathways, most prominent of which is induction of telomerase leading to immortalization of mammary epithelial cells [46]. d) Downregulation of Bmi-1 leads to de-repression of ink4a, which encodes tumor suppressors p16Ink and p19Arf that regulate senescence and apoptosis [47]. Activation of these pathways have been invoked as an important tumor suppressor barrier, because these pathways act as potent inhibitors of proliferation or propagation of damaged cells. e) In addition we posit that cisplatin and Bmi-1 act on similar pathways affecting mitochondrial function and/or through increased ROS generation cause DNA damage leading to efficient induction of apoptosis. Increased levels of DNA damage then augment the signal initiating apoptosis. Thus, Bmi-1 is an important new target for therapy not only in chemoresistant ovarian cancer but also for other malignancies characterized by overexpression of Bmi-1.

## Materials and Methods

### Reagents

Bmi-1 antibody was from Zymed, CA, USA. Bmi-1 and scrambled control siRNA were from Sigma-Aldrich Sigma-Aldrich, St. Louis, MO. Phospho-H2AX, Phospho-Chk-2, 53 BP1 cleaved caspase-8, caspase-9 and PARP antibodies were from Cell Signaling Technologies Inc., MA. Annexin/FITC-PI apoptosis kit was from Biovision Inc.

### Cell Culture

A-2780 and CP-70 cells were grown in RPMI with 10% FBS and 1% antibiotic (Penicillin/Streptomycin) according to the provider's recommendation. The CP-20 cell lines were grown according to our previously published procedures [48].

### SiRNA transfection

The ovarian cells A2780 or CP-70 were grown in their respective medium for one day prior to transfection. Using oligofectamine the cells were transfected with 25 nM scrambled control or Bmi-1 siRNA. After 48 h the cells were processed for western blot or apoptosis assays [49].

### Western Blot

Harvested ovarian cancer cells, both treated and non-treated, were washed in PBS and lysed in ice-cold radioimmunoprecipitation (RIPA) buffer with freshly added 0.01% protease inhibitor cocktail (Sigma) and incubated on ice for 30 min. Cell debris was discarded by centrifugation at 13000 rpm for 10 min at 4°C and the supernatant (30–50 µg of protein) was run on an SDS-Page [49].

### ROS assay

The ovarian cancer cell lines transfected with the scrambled control or Bmi-1 siRNA were treated with cisplatin for 24 hrs. After washing with HBSS, the cells were incubated with 5 µM carboxy-H2DCFDA (Invitrogen, Carlsbad, CA) [50] in fresh HBSS for 30 min at 37°C. Excessive probe was washed off. The cells were harvested with trypsin and fluorescence of the labeled cells was measured at an excitation wavelength of 485 nm and emission wavelength of 530 nm by using Fluorolog 3 (Jobin-Yvon Horiba). The experiment was repeated three times and mean fluorescence intensity (MFI) [50] was recorded. Statistical significance was determined using two-sided Student's t test, and P<0.05 was considered significant.

### Apoptosis assay

Ovarian cancer cells transfected with scrambled control or Bmi-1 siRNA for 48 h were treated with cisplatin for another 48 h. For experiments using NAC, it was applied 1 h before addition of cisplatin. Cells were subjected to Annexin-FITC/PI staining and flourescence recorded using a FACSCalibur flow cytometer (Becton-Dickinson). The experiment was performed in triplicate and significance determined using two-sided Student's t test, P<0.05 was considered significant.

### GSH assay

The assay was performed according to manufacturer's protocol (Cayman Chemicals). Briefly, the ovarian cancer cell lines transfected with the scrambled control or Bmi-1 siRNA were treated with cisplatin for 24 hrs. The cell were lysed by sonication and collected in 50 mM phosphate buffer by centrifugation at 10,000 g for 15 min at 4°C. The lysates were next de-proteinated using the TEAM reagent and total cellular GSH determined against a standard curve generated at the same time by measuring absorbance at 405 nm 25 min after addition of assay cocktail. The experiment was performed in triplicate and significance determined using two-sided Student's t test, P<0.05 was considered significant.

### Realtime PCR

Total RNA was isolated from transfected cells using TRIzol reagent (Invitrogen). RNA was first retrotranscribed using TaqMan® Reverse Transcription Kit (Applied Biosystems) and then realtime PCR was carried out using and TaqMan® SYBR Green Master Mix (Applied Biosystems). The primers for human Bmi-1, GCLM, GSS and beta actin were from SA Bioscienes, Frederick, MD. The comparative C_t_ method was used to calculate the relative abundance of mRNA compared with that of beta actin expression [51]. The experiment was performed in triplicate and significance determined using two-sided Student's t test, P<0.05 was considered significant.

### Liposomal siRNA preparation

For in vivo delivery, siRNA was incorporated into DOPC as previously described [48]. Briefly, siRNA and DOPC were mixed at a ratio of 1∶10 (w/w) siRNA/DOPC in excess tertiary butanol. Tween 20 was added to the mixture at the ratio of 1∶19 (Tween 20:siRNA/DOPC). After vortexing, the mixture was frozen in an acetone/dry ice bath and lyophilized. Before in vivo administration, this mixture was hydrated with 0.9% saline to a concentration of 25 µg/mL and 200 µL of mixture were used per injection.

### Orthotopic model of ovarian cancer

Female athymic nude mice (NCr-nu) were purchased from the National Cancer Institute-Frederick Cancer Research and Development Center (Frederick, MD). All mice were housed and maintained under specific pathogen-free conditions in facilities approved by the American Association for Accreditation of Laboratory Animal Care (Acuf# 12-02-18233) and in accordance with current regulations and standards of the U.S. Department of Agriculture, U.S. Department of Health and Human Services, and NIH. All studies were approved and supervised by the University of Texas M. D. Anderson Cancer Center Institutional Animal Care and Use Committee. All mice were used in these experiments when they were 8 to 12 wk old.

Before injection, tumor cells were washed twice with PBS, detached by 0.1% cold EDTA, centrifuged for 7 min, and reconstituted in HBSS (Invitrogen). Cell viability was confirmed by trypan blue exclusion. Tumors were established by i.p. injection of either 1.0×10^6^ CP20 cells. Once established, this tumor model reflects the growth pattern of advanced ovarian cancer [42].

To assess the effects of siRNA therapy on tumor growth, treatment was initiated 1 wk after i.p. injection of tumor cells. Mice were divided into four groups (n = 10 mice per group): (a) control siRNA-DOPC (150 µg/kg i.p. twice weekly), (b) control siRNA-DOPC + cisplatin (160 µg/mouse i.p. weekly), (c) Bmi-1 siRNA-DOPC (150 µg/kg i.p. twice weekly), and (d) Bmi-1 siRNA-DOPC + cisplatin (doses same as individual treatments). Treatment was continued until 4 weeks after tumor inoculation. At the time of sacrifice, mouse weight, tumor weight, number of nodules, and distribution of tumors were recorded. Tissue samples were snap frozen for lysate preparation or fixed in formalin for paraffin embedding. The individuals who did the necropsies, tumor collections, and tissue processing were blinded to the treatment group assignments.

### Immunohistochemistry

Ki67 and terminal deoxynucleotidyl transferase–mediated dUTP nick end labeling (TUNEL) staining were done using formalin-fixed, paraffin-embedded tumor sections (8 µm thickness) as previously described [48]. Briefly, after deparaffinization and rehydration, antigen retrieval was done using citrate buffer (0.1 mol/L; pH 6.0) in a microwave. Endogenous peroxidase and nonspecific epitopes were blocked with 3% H2O2/methanol for 12 min and 5% normal horse serum and 1% normal goat serum for 20 min. Sections were incubated with primary anti-Ki67 overnight at 4°C and secondary horseradish peroxidase–conjugated antibody (Serotec Bioproducts) for 1 h at room temperature. Horseradish peroxidase was detected with 3,3′-diaminobenzidine (Phoenix Biotechnologies) substrate for 5 min, washed, and counterstained with Gill's no.3 hematoxylin (Sigma-Aldrich) for 15 s and mounted.

To quantify apoptosis, we did TUNEL staining on 8-µm-thick paraffin-embedded tumor slides as previously described [48]. Briefly, after deparaffinization, slides were treated with proteinase K (1∶500) and a positive control slide was treated with DNase. Endogenous peroxidase activity was blocked with 3% H2O2 in methanol. After being rinsed with TdT buffer (30 mmol/L Trizma, 140 mmol/L sodium cacodylate, 1 mmol/L cobalt chloride), slides were incubated with terminal transferase (1∶400; Roche Diagnostics) and biotin-16-dUTP (1∶200; Roche Diagnostics) and blocked with 2% bovine serum albumin. Slides were then incubated with peroxidase streptavidin (1∶400) at 37°C for 40 min, visualized with 3,3′-diaminobenzidine chromogen, and counterstained with Gill's hematoxylin. The apoptotic and proliferative indices were determined by the number of positive cells in five randomly selected high-power fields exclusive of necrotic areas. To quantify Ki67 expression and apoptotic cells, the number of positive cells (3,3′-diaminobenzidine staining) was counted in 10 random 0.159 mm2 fields at ×100 magnification. All staining was quantified by two investigators in a blinded fashion.

### Statistical analysis

All values are expressed as means±SD. Statistical significance was determined using two-sided Student's t test, and a value of P<0.05 (*) was considered significant.

For animal experiments, 10 mice were assigned per treatment group. To judge the necessary sample size for proposed experiments, we considered a two-way ANOVA model. For an effect size (ratio of fixed effect and residual SD) of 1.3, this sample size will be sufficient to provide 80% power for a test at significance level of 0.05. Mouse and tumor weights and the number of tumor nodules for each group were compared using Student's t test (for comparisons of two groups). Statistical analyses were done using Statistical Package for the Social Sciences 12.0 for Windows (SPSS, Inc.). A two-tailed P≤0.05 was deemed statistically significant.
